# Electropneumatic system for the simulation of the pulmonary viscoelastic effect in a mechanical ventilation scenario

**DOI:** 10.1038/s41598-023-41881-0

**Published:** 2023-12-02

**Authors:** Jacobo Castaño, Mario A. Giraldo, Yesid Montoya, Yeison J. Montagut, Andrés F. Palacio, León D. Jiménez

**Affiliations:** 1 Universidad EIA, Envigado, Colombia; 2 Hospital Alma Máter de Antioquia, Medellín, Colombia

**Keywords:** Respiration, Biomedical engineering, Electrical and electronic engineering, Mechanical engineering

## Abstract

The viscoelastic properties of the lung have important implications during respiratory mechanics in terms of lung movement or work of breathing, for example. However, this property has not been well characterized due to several reasons, such as the complex nature of the lung, difficulty accessing its tissues, and the lack of physical simulators that represent viscoelastic effects. This research proposes an electropneumatic system and a method to simulate the viscoelastic effect from temporary forces generated by the opposition of magnetic poles. The study was tested in a mechanical ventilation scenario with inspiratory pause, using a Hamilton-S1 mechanical ventilator (Hamilton Medical) and a simulator of the human respiratory system (SAMI-SII). The implemented system was able to simulate the stress relaxation response of a Standard Linear Solid model in the Maxwell form and showed the capacity to control elastic and viscous parameters independently. To the best of our knowledge, this is the first system incorporated into a physical lung simulator that represents the viscoelastic effect in a mechanical ventilation scenario.

Since the crucial role of elastic recoil in respiration became known, the mechanical properties of the lung have been a focus of interest in the scientific community^1^. The viscoelastic properties of the lung have important implications during respiratory mechanics in terms of lung deflation^2^, frequency dependence of elastance and resistance^3^, respiratory work^4^, and performance of forced expiratory vital capacity maneuvers^5^. In mechanical ventilation, the inspiratory pause is a technique that involves the occlusion of airflow when the entire tidal volume enters the airway, which generates an absence of airflow for a few seconds and finishes with the opening of the exhalation valve. It is thought that this technique favors more homogeneous ventilation by allowing the redistribution of gases in the alveoli. Also, it is used to study ventilatory mechanics and to obtain information about lung conditions^6^. Since 1980, the inspiratory pause technique in mechanical ventilation has made it possible to study the effect of viscoelasticity in cats^7^, dogs^8–10^, and healthy humans^4,11^, which has allowed the development of mathematical models that have approximated the behavior of these properties. These studies showed that the total resistance of the respiratory system (R_rs_) could be split into a resistance associated with the airway (R_aw_) and a resistance given by the dissipation of viscoelastic pressure within the lung and the chest wall (ΔR). Thus, when an inspiratory pause occurs during mechanical ventilation, there is an immediate pressure drop associated with R_aw_ and then another exponential drop associated with ΔR^12^. This last drop in pressure is a phenomenon called stress relaxation and it is manifested by the constant deformation of viscoelastic materials.

Mechanical modeling is a tool that has been extensively used to describe the viscoelastic behavior of the lungs^13–15^. Mechanical representations of viscoelasticity are usually based on elastic (springs) and viscous components (dashpots), which lead to having both solid and fluid phases^16^. Maxwell and Kelvin Voigt models are the simplest representation of a viscoelastic material, Maxwell model places a spring and a dashpot in series while Kelvin Voigt model places them in parallel. More complex models have been developed such as Weichert and fractional order derivative Standard Linear Solid (FSLS), which have been compared with viscoelastic tissue mechanical behavior^13,17^. The simplest model capable of representing stress relaxation and creep is the Standard Linear Solid (SLS), which has been used to characterize pig liver and spleen viscoelastic response^18^, to represent blood vessels viscoelasticity for modeling blood flow^19^ and to describe calf heart stress relaxation response^20^. Regarding pulmonary biomechanics, the model has been employed to represent human lung fibroblasts to know the effect of mechanical stimulation^21^ and to describe parenchyma behavior^14^, which has also yielded acceptable fits in the curves of stress relaxation of pig lungs^13,15^.

Despite progress, the understanding that links the structure of lung tissues and their function remains incomplete^1^, as such pulmonary viscoelasticity has not been completely characterized. Although it is mentioned as a potential biomarker, studies have suggested that further research is required^10,13^. The lung is an organ that cannot be understood based on its isolated properties, but it is necessary to see how its constituents are organized among themselves^1^. Also, the pulmonary hermeticity and its complex dynamics make its study difficult. Analogously, the use of respiratory mechanics simulators that represent the viscoelastic properties may yield a better understanding of different lung scenarios and the role of viscoelasticity in pathological and healthy conditions; however, there are currently no simulators with such features.

In the present study, an electropneumatic system was developed to be coupled to a physical simulator of human respiratory biomechanics whose objective is to give the simulator the capacity of reproducing the viscoelastic effect. With this aim, two factorial designs were performed in a mechanical ventilation scenario which allowed the calculation of the parameters of an SLS model in the Maxwell form and to make a comparison of the stress relaxation curves obtained experimentally and with the model.

## Results

### Electropneumatic system for viscoelastic simulation

**Figure 1:**
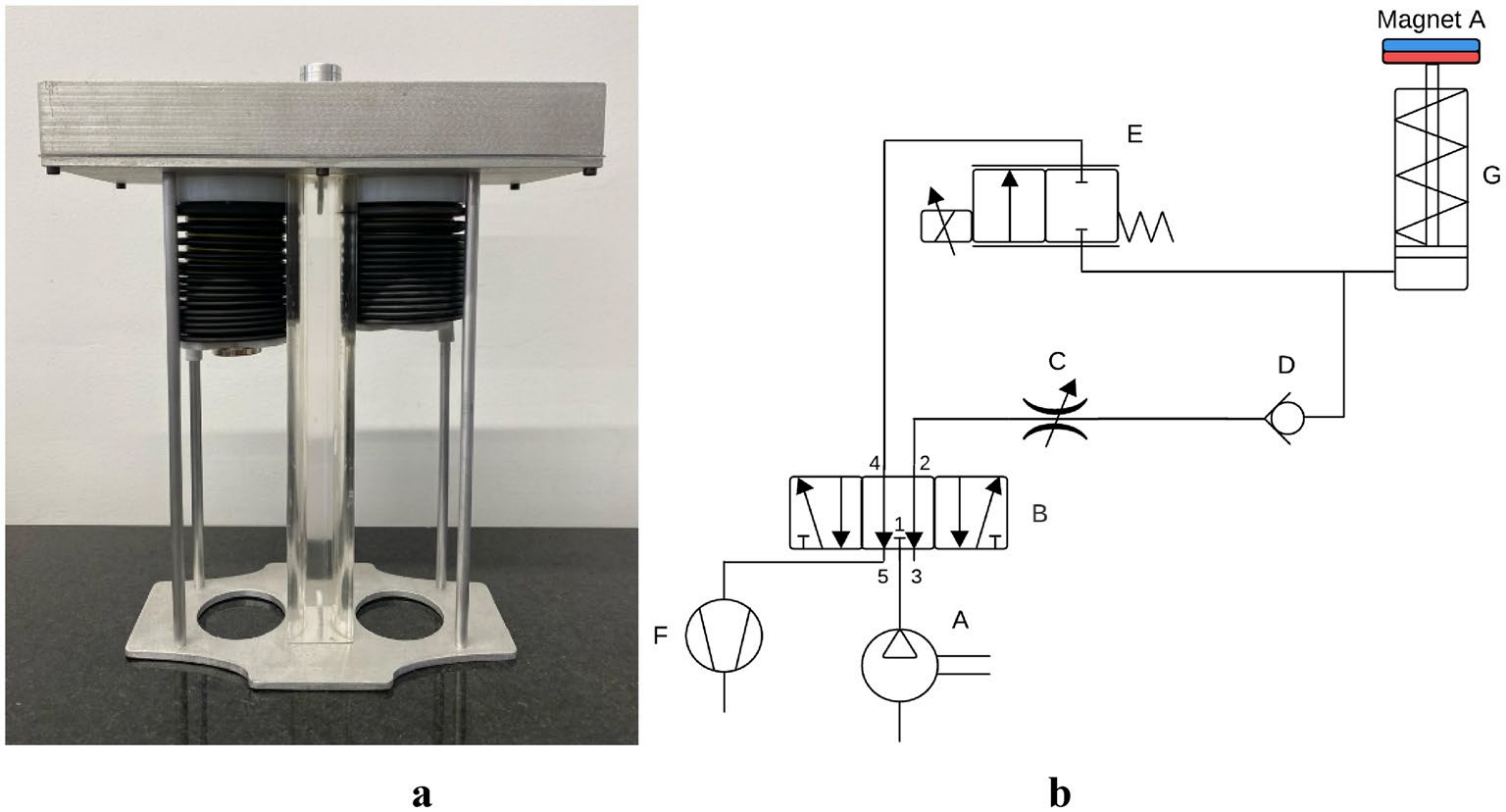
(**a**) alveolar unit of SAMI-SII. It is composed of two bellows interconnected with each other through a tube that represents the airway and allows the connection to a mechanical ventilator. (**b**) Pneumatic module schematic.

A Hamilton-S1 mechanical ventilator (Hamilton Medical, Switzerland) was used to establish the mechanical ventilation scenario. This was connected to an alveolar unit of the SAMI-SII respiratory biomechanics simulator (Fig. 1a). SAMI-SII allows the simulation of pathological and healthy lung conditions by modifying two biomechanical variables: airway resistance and lung compliance. The resistance is controlled by adjusting the diameter of the tubes that transport the air into the SAMI-SII (the narrower the diameter, the greater the resistance). Compliance is modeled with springs, which are tied to each of the bellows. The modular design of the system provides the flexibility to configure distinct compliances and resistances for the alveolar units, enabling the simulator to accurately emulate diverse lung scenarios.

An electropneumatic system was built to generate the viscoelastic effect in the alveolar unit of SAMI-SII. The pneumatic module is shown in Fig. 1b. A compressor was used to supply air at a pressure of 55 psi (*A*). Before each experiment, the pressure was checked with a VT650 Gas Flow Analyzer (Fluke Biomedical, U.S.). The compressor was connected to port 1 of a 5/3 solenoid valve (*B*). To port 2, an adjustable flow regulator (*C*) and a check valve (*D*) were connected in series. The flow regulator was used to control the speed of the extension stroke of a single-acting pneumatic cylinder (*G*) and avoid abrupt displacements of the rod. For the return stroke, a proportional flow control valve (*E*) and a vacuum pump (*F*) were used. An Arduino UNO board was used to control the pneumatic module. The solenoid valve (*B*) and the vacuum pump (*F*) were controlled through digital outputs of the microcontroller. The proportional valve (*E*) was controlled with a 490 Hz PWM output and a 24 V power supply. Although the solenoid valve had three potential positions, only two were used, as the control of one of its two solenoids was sufficient for the intended purpose.

**Figure 2:**
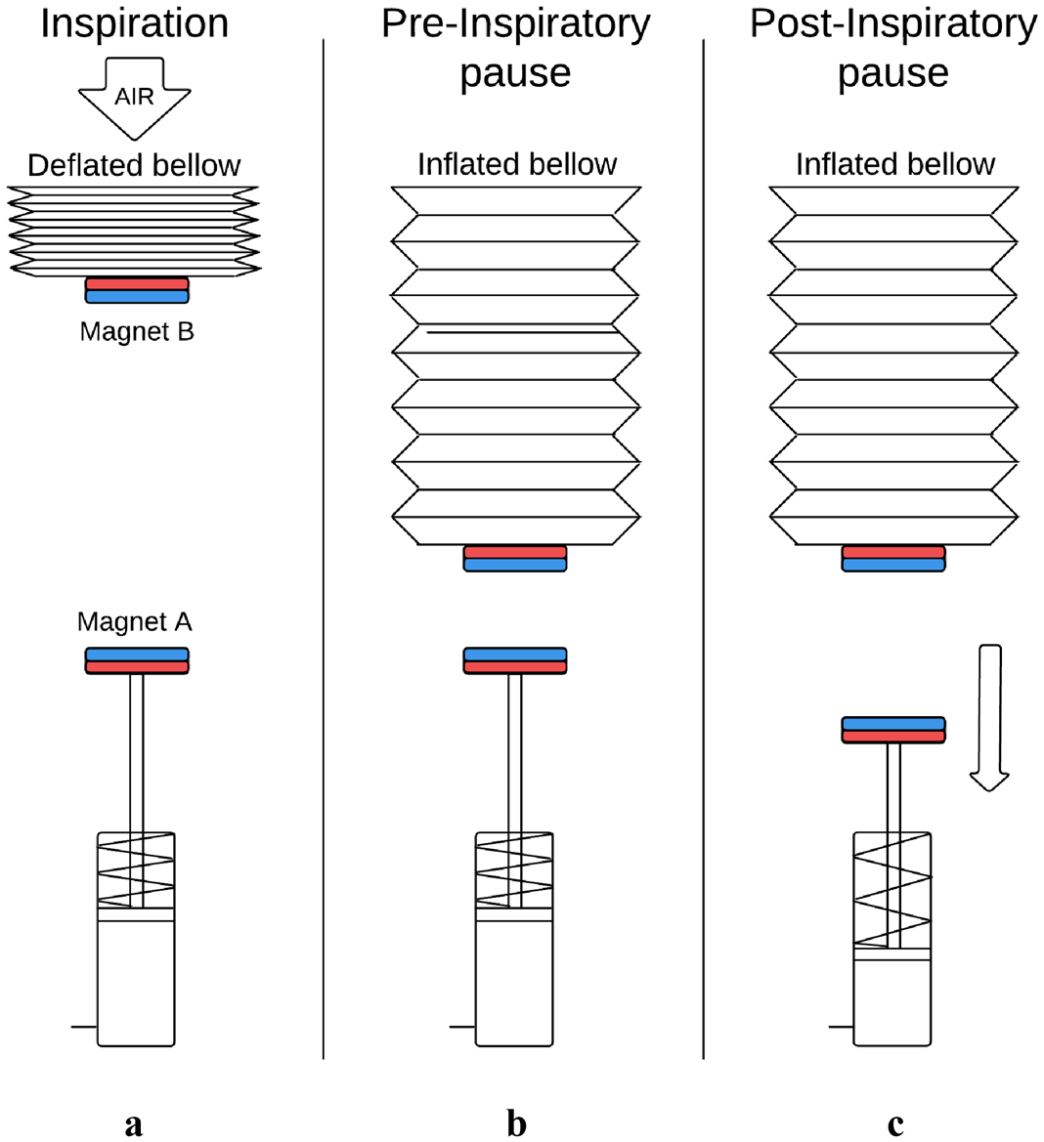
(**A**) The rod of the pneumatic cylinder is extended during inspiration. (**B**) The mechanical ventilator has already filled the bellow of the alveolar unit with the programmed tidal volume and an inspiratory pause is about to occur. The rod is still extended. (**C**) At the moment of inspiratory pause, the return stroke occurs.

**Figure 3:**
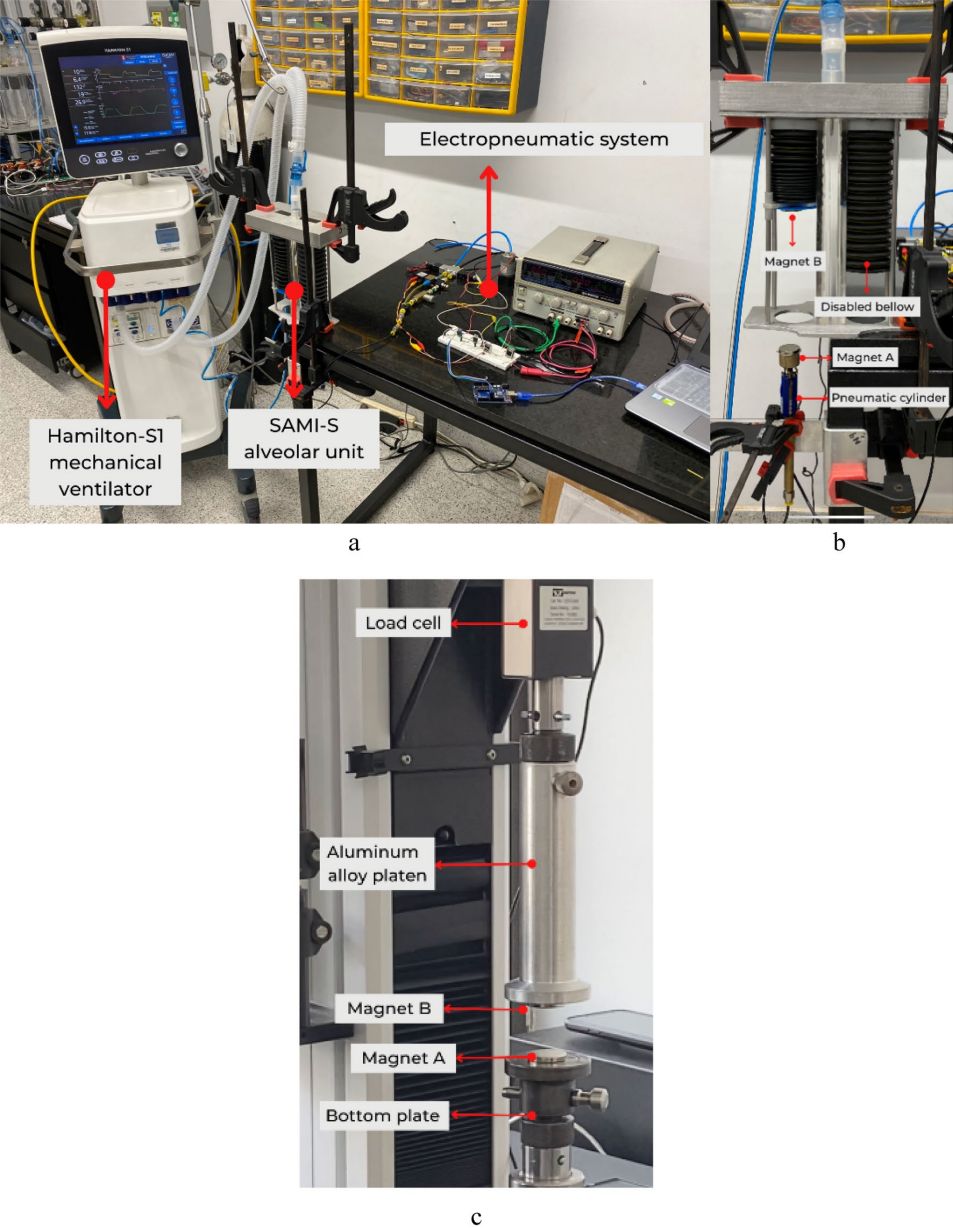
The mechanical ventilation scenario in which the viscoelastic effect was simulated. (**a**) Hamilton-S1 mechanical ventilator, SAMI-SII alveolar unit, and electropneumatic system are shown. (**b**) SAMI-SII alveolar unit and the single-acting pneumatic cylinder is shown with their respective magnets (the bellow on the right was disabled for the experiments). (**c**) Experimental setup for magnets characterization in the Instron load system 3345.

The strategy employed for simulating the effect of viscoelasticity in mechanical ventilation is based on the repulsion force generated between two magnets. One of the magnets (*Magnet A*) was attached to a pneumatic cylinder rod and the other (*Magnet B*) to the bottom of the bellow of the alveolar unit in such a way that both magnets faced the same poles (Fig. 2). In this position, forces are generated by the repulsion of the magnetic fields that cause an increase in the internal pressure of the bellows (an additional pressure to the one generated by the mechanical ventilator). During each inspiration, the rod was extended (Fig. 2A). At the moment of inspiratory pause, the return stroke occurred to move *Magnet A* downwards (Fig. 2C). By changing the distance between the magnets and the speed of the return stroke, this configuration allowed to simulate mechanical ventilation scenarios with different viscoelastic responses. Figure 3a and b show the resulting experimental setup in which the viscoelastic effect was simulated.

### Magnets characterization

Before conducting the mechanical ventilation tests, a magnet characterization was undertaken to describe the relationship between the repulsion force and the distance between the magnets (AB Distance). In the case of *Magnet B*, a trio of distinct magnets was subjected to testing: a neodymium magnet with a thickness of 4 mm, another neodymium magnet with a thickness of 2 mm, and a ceramic magnet with a thickness of 4 mm (all magnets had a 25 mm diameter). Throughout all experiments, a 4 mm thick neodymium magnet served as *Magnet A*. Additionally, this force was described in the time domain by moving the magnets away at different speeds (30 mm/min, 50 mm/min, 80 mm/min). For this, both *Magnet A* and *Magnet B* were 4 mm thick neodymium magnets. These experiments were performed using an Instron load system 3345 (Instron, U.S.) (Fig. 3c).

**Figure 4:**
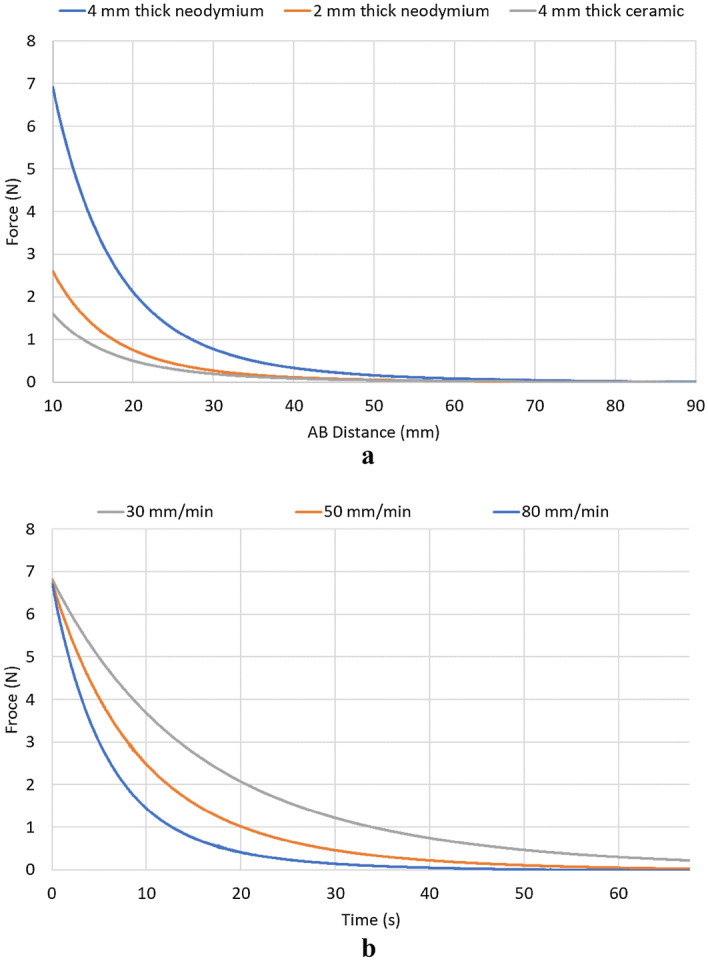
(**a**) Relationship between repulsion force of magnets and AB Distance. Blue, orange, and gray curves correspond for 4 mm thick neodymium, 2 mm thick neodymium, and 4 mm thick ceramic magnets for *Magnet B*, respectively. For *Magnet A*, a 4 mm neodymium magnet was set. (**b**) Time domain representation of the repulsion force of magnets by moving them away at different speeds. Blue, orange, and gray curves correspond for 80 mm/min, 50 mm/min, and 30 mm/min, respectively.

Figure 4a shows the resultant relationship curves between the repulsion force generated by the different magnets used and AB Distance. When AB Distance was 10 mm, the force generated was 6.91 N, 2.60 N, and 1.59 N. On the other hand, Fig. 4b shows the resulting curves from moving the magnets at different speeds (In this case, just 4 mm thick neodymium magnets). The higher the speed, the faster the force gets closer to 0 N. For example, after 10 s, the force decayed 46.74%, 64.25%, and 79.16% for a speed of 30 mm/min, 50 mm/min, and 80 mm/min, respectively.

### Viscoelastic simulation in the mechanical ventilation scenario

A volume-controlled mechanical ventilation (CMV) scenario was simulated using the Hamilton-S1 mechanical ventilator on the SAMI-SII. 4 mm thick neodymium magnets were employed. Two types of experimental designs known as the 2^2^-factorial design (2 factors with 2 different levels) and the 2^4^-factorial design (4 factors with 2 different levels) were executed. These designs efficiently allow analyzing how different factors interact and influence a dependent variable, a crucial aspect for comprehending the relationships among ventilation parameters in the experiments.

For the 2^2^-factorial design, the factors considered were the PWM (68.63%, 74.50%) and AB Distance (25 mm, 30 mm). The remaining ventilation parameters were as follows: respiratory rate of 13 rpm, R_aw_ of 18 cmH2O/L/s, tidal volume of 150 mL, and inspiratory pause of 35%. This design was also run three times, for a total of 12 executions.

On the other hand, the 2^4^-factorial design was implemented to explore the interaction of four different factors: R_aw_ (5 cmH2O/L/s, 8 cmH2O/L/s), compliance (15 mL/cmH2O, 20 mL/cmH2O), tidal volume (100 mL, 150 mL), and AB Distance (25 mm, 30 mm). Each factor was assessed at two different levels within this design. Apart from these factors, other parameters remained constant, such as a respiratory rate of 13 rpm, an inspiratory pause of 20%, and the PWM controlling the proportional valve set at 70%. This design was run three times, resulting in a total of 48 executions. Through this design, the aim was to analyze how these four variables combined and affected the dependent variable, thereby providing detailed insight into the intricate interactions among factors in the context of mechanical ventilation.

In both experimental designs, the dependent variable was the pressure inside the bellow, which was measured with the mechanical ventilator and recorded using a Hamilton Datalogger (Hamilton Medical, Switzerland).

**Figure 5:**
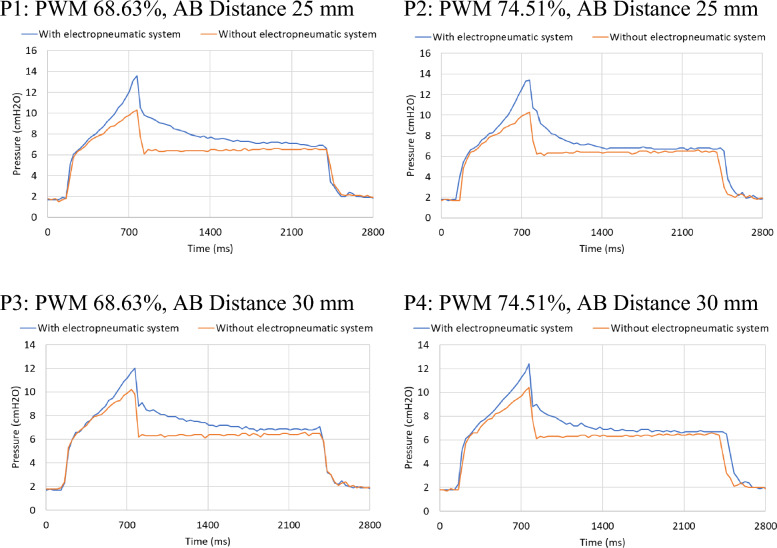
Pressure curves obtained from the mechanical ventilation scenario using and without using the electropneumatic system to simulate the viscoelastic effect. The different combinations of AB Distances and PWM given by the 2^2^-factorial design are shown.

Figure 5 shows a single cycle from one of the runs within the 2^2^-factorial design. When using the electropneumatic system, there is an increment in the peak pressure, followed by a rapid pressure drop when an inspiratory pause occurs, and then the pressure drops slowly to an asymptotic plateau value. Conversely, when the electropneumatic system was not employed, the paused pressure drop is not observed. At the moment of inspiratory pause, the pressure drops immediately to the plateau value.

### Model parameters calculations

**Figure 6:**
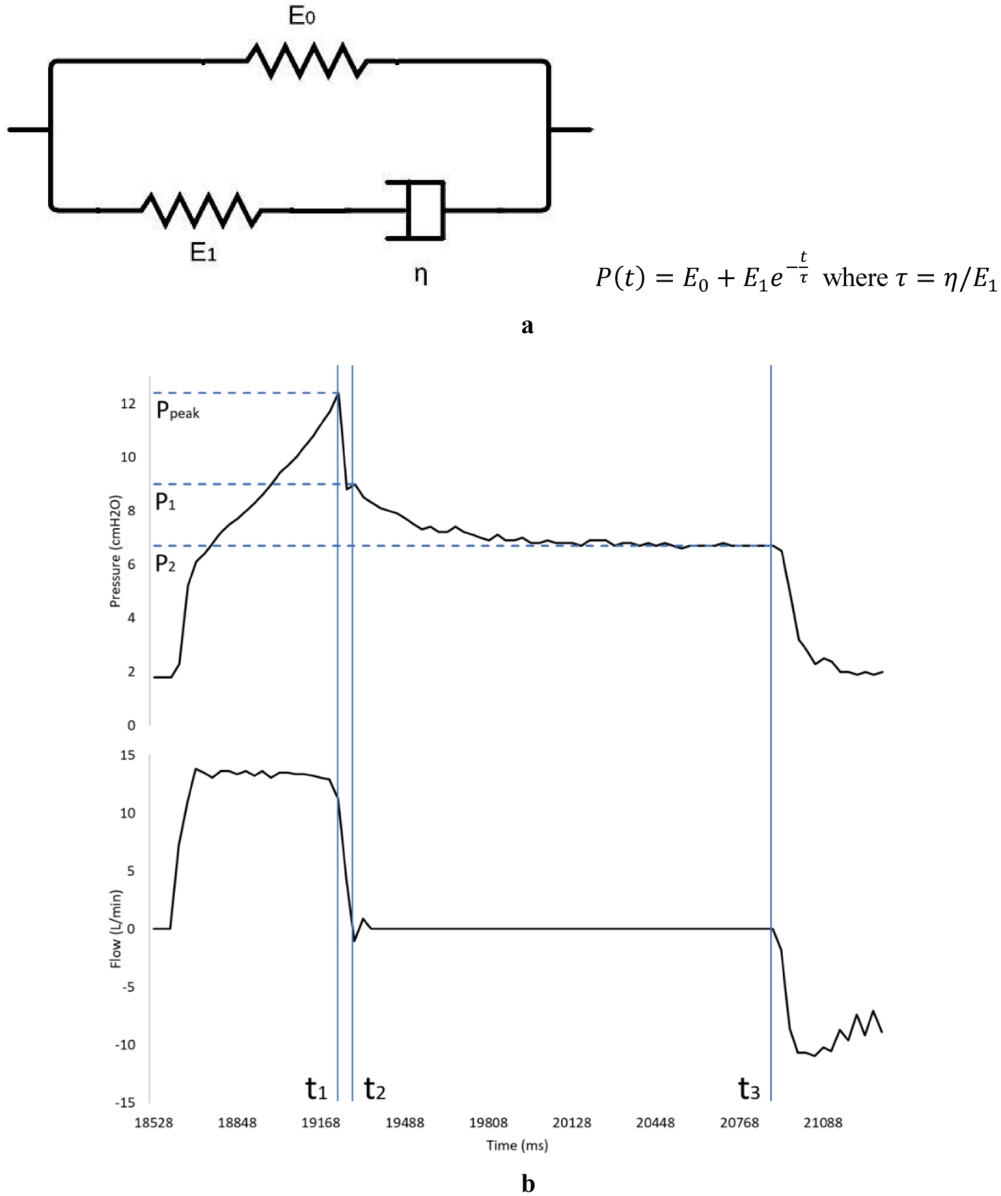
(**A**) SLS discrete element rheological model schematic and its governing equation. (**B**) One cycle of the pressure and flow curve obtained from the mechanical ventilation scenario with an inspiratory pause with 74,51% PWM and 30 mm AB Distance. At t_1_ inspiratory pause occurs and at t_2_ flow becomes zero. P_1_ is the pressure when the flow is zero. P_2_ is the plateau pressure. P_peak_-P_1_ is because of airway resistance and P_1_ − P_2_ is given by the effect of the cylinder return stroke.

Figure 6A shows the discrete element rheological model schematic of the SLS model with its respective governing equation, where *E*_*n*_ symbolizes the modulus of elasticity of each spring and *η* the viscous constant of the dashpot. Regarding lung biomechanics, *E*_*0*_ represents the elastic solid part of the respiratory system tissue, essentially dictating its overall compliance. *E*_*1*_ and *η* represent the fluid part of the respiratory system tissue, symbolizing the elastic fibers that release their stress during the inspiratory pause, causing the stress relaxation phenomenon. SLS parameters were calculated with the resulting data of the experiments.

From the 2^4^-factorial design, the pressure decay generated solely by the return stroke of the cylinder (P_1_-P_2_) was calculated as Fig. 6B shows (ignoring the pressure drop generated by airway resistance). The averaged P_1_-P_2_ drops measured 3.48 ± 0.22 cmH2O and 2.20 ± 0.17 cmH2O for AB Distances of 25 mm and 30 mm, respectively. The D’Agostino-Pearson test demonstrated that the P_1_-P_2_ data conformed to a normal distribution at a significance level of α = 0.05, with a p-value of 0.44 for 25 mm and 0.37 for 30 mm. Consequently, a hypothesis test was conducted utilizing a one-sample t-test with α = 0.05, yielding p-values of 0.999 for 25 mm and 0.981 for 30 mm. Thus, *E*_*1*_ was found to be 3.48 cmH2O and 2.20 cmH2O for an AB Distance of 25 mm and 30 mm, respectively.

**Figure 7:**
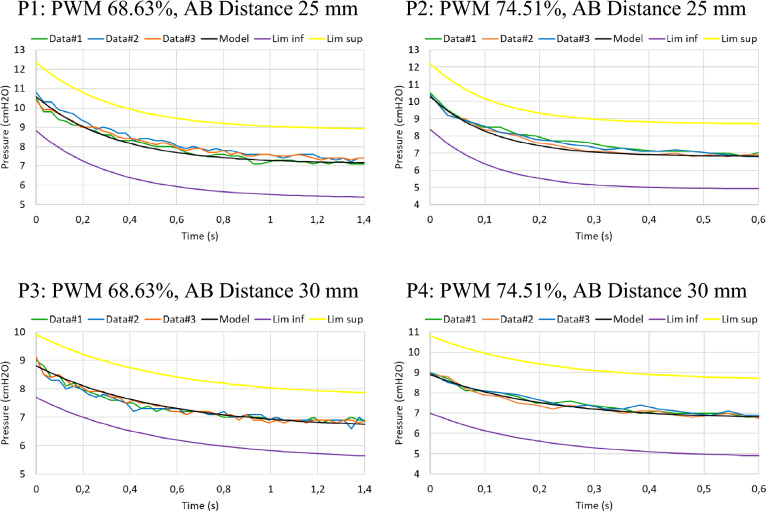
Slow pressure drop sample data after inspiratory pause compared to the stress relaxation response of an SLS model in the Maxwell form, with a 95% confidence interval. The different combinations of AB Distances and PWM given by the 2^2^-factorial design are shown.

The paused pressure drops curves from 2^2^-factorial design were employed to conduct an exponential regression to find the value of *η* and *E*_*0.*_ In Fig. 7, the sample data from the slow pressure drop compared to the stress relaxation response of the SLS model in the Maxwell form is shown; besides a 95% confidence interval. The consistent decreasing trend shared by both the model and the experimental data signifies a pronounced alignment of the proposed model. The R^2^ values were 96.02%, 96.79%, 95.51%, 96.06% for P1, P2, P3 and P4, respectively (Table 1). This model predicts a fast decrease of pressure at the beginning of inspiratory pause followed by an asymptotic plateau value as usually is seen in clinical scenarios^4,11^. Table 1 compiles the resulting parameter values of the model.

**Table 1:** Values of parameters obtained from fitting the SLS model in the Maxwell form to the mechanical ventilation scenario data.

	P1	P2	P3	P4
AB Distance (mm)	25	25	30	30
PWM	68.63%	74.51%	68.63%	74.51%
R^2^	96.02%	96.79%	95.61%	96.06%
*E*_*0*_* (cmH*_*2*_*O)*	7.0999	6.7999	6.5999	6.6999
*η (cmH*_*2*_*0·s)*	1.181	0.403	1.166	0.442
*E*_*1*_* (cmH*_*2*_*O)*	3.48	3.48	2.20	2.20

## Discussion

In this study, an electropneumatic system was tested to represent the lung viscoelastic effect using a respiratory biomechanics simulator in a mechanical ventilation scenario. The system showed the ability to simulate the stress relaxation response of an SLS model in the Maxwell form, which has been widely used to describe the viscoelastic effect of lung tissue.

The characterization of the magnets describes the behavior of the force generated by the repulsion of its poles under diverse conditions, to understand the possible effects that would be reflected using the electropneumatic system in the mechanical ventilation scenario. The force's magnitude depends on factors such as the magnet's material and dimensions, as these elements influence modifications in its magnetic field. Based on the technique that is being used to simulate viscoelasticity, these results propose the feasibility of modulating the pressure drop's magnitude, induced by the viscoelastic system, through the utilization of distinct magnets and/or alterations in the AB Distance (Fig. 4a). Furthermore, the greater the separation speed between the magnets, the faster the force falls (Fig. 4b). This observation holds the potential to govern the specific timing at which the pressure drops within the bellows, thereby offering a prospect for fine-tuned control over this pivotal aspect of the system.

The behavior of the lung as viscoelastic material is mainly given by elastin (which contributes to elasticity) and proteoglycans (in charge of its viscous characteristics by stabilizing the elastin and collagen network) of the extracellular matrix. This composition empowers lung tissue with the ability to “accommodate” stress after volume changes (stress relaxation)^15,22^. Viscoelasticity makes lung mechanics time-dependent, and this behavior can be observed in the pressure–time curve by abruptly stopping the inspiratory flow, which results in a slow decrease in pressure as a reflection of a conformational adaptation of the parenchymal fiber. Pulmonary stress is proportional to strain and strain rate, both of which are intrinsically linked to the lung's viscoelastic traits. Consequently, variations in respiratory rate, while maintaining the same tidal volume, can lead to changes in pulmonary stress levels^23^. An alteration in the viscoelastic characteristics of the lung has been described in animal models when pulmonary fibrosis is induced in them (a condition characterized by an excess in the production of extracellular matrix molecules such as collagen, elastin, and proteoglycans), generating an increase in elastance and a decrease in hysteresivity^22^. In addition to pulmonary fibrosis, other pathologies such as asthma and emphysema have been identified, which are related to significant changes that can be local or diffuse in lung viscoelasticity^24^.

The SLS model in the Maxwell form exhibits viscoelastic behavior in its elastic and viscous phases. *E*_*0*_ represents the modulus of elasticity of the elastic part of the solid, which according to the results, it takes an asymptotic value at plateau pressure (Table 1), aligning with other studies carried out^13,25^. This is because the plateau pressure is a parameter that depends on lung compliance. Consequently, the value of *E*_*0*_ within the model corresponds to the modulus of elasticity inherent to the spring employed inside the bellows.

The fluid part of the solid is represented by *E*_*1*_ and *η*, denoting the modulus of elasticity and the viscosity, respectively. *E*_*1*_ shows a strong indication (α = 0.05) that it is given by AB Distance, a determinant of the magnitude of the pressure drop following the inspiratory pause (When the mechanical ventilator´s airflow is zero)^25^. This pressure drop results from the pneumatic cylinder's return stroke, inducing the magnets to distance themselves. This spatial shift causes the repulsion force between them to disappear, reducing the internal bellows pressure. Moreover, the results indicate that *η* is given by the speed of the return stroke, regulated by the PWM (Table 1). The PWM modifies the flow that passes through the proportional valve, thereby influencing the time taken for the pneumatic cylinder to evacuate the air chamber and execute the return stroke. Hence, the greater the PWM applied, the faster the rod moves downwards, increasing the velocity of the pressure drop. From the regression analyses performed, the effect of the internal pressure of the bellows after the inspiratory pause generated by the electropneumatic system had a strong tendency (α = 0.05) to behave like the SLS model in the Maxwell form in its stress relaxation response (Fig. 7). All the coefficients of determination R^2^ were greater than 95%, evidence of the robustness of the model's predictive capability (Table 1).

The results suggest that the implemented electropneumatic system can represent the pulmonary viscoelastic effect in a mechanical ventilation scenario and that it can modify its elasticity and viscosity parameters independently by adjusting AB Distance and the PWM, respectively, which agrees with the magnets characterization conducted with the load system.

The proposed electropneumatic system carries a distinctive advantage in its capability to independently manipulate viscosity ($\eta )$ and the modulus of elasticity $({E}_{1})$ independently. This flexibility permits the establishment of diverse relationships between the elastic and viscous phases, giving the possibility to control $\tau$ ($\tau =\eta /{E}_{1}$), a factor to determine the desired pressure drop rate. Another advantage lies in the mechanism driving pressure generation within the bellows through magnetic repulsion, which presents a system of low friction and inertia, avoiding effects and forces that could alter the good performance of the system. The lung is an organ with low inertia and friction that works at low pressures, so when simulating a ventilation scenario, it is important to ensure that these effects are minimal if there is no control over them^26^. The operational principle could be extrapolated to various physical simulators, contingent upon an investigation to discern the necessary adaptations required to implement the technique at hand. Although the behavior of the system fits acceptably with the SLS model in its stress relaxation response, it would be pertinent to undertake further comparisons with different models such as the FSLS, which has been used frequently and has shown better adjustments in the behavior of different viscoelastic tissues^13–15^. It is known from different research that the properties of the lung are dependent on the respiratory frequency^3,14,27^; however, it is important to note that this study's scope is confined to evaluating the electropneumatic system's performance at a singular frequency, so it would be convenient to conduct an exploration to find out the viscoelastic response of the system in a wider frequency range.

To the best of our knowledge, this is the first system incorporated into a physical lung simulator that represents the viscoelastic effect in a mechanical ventilation scenario. The system showed the ability to simulate the stress relaxation response of an SLS model in the Maxwell form, which has been widely used to describe the behavior of viscoelastic biological tissues. Future work may take into consideration the method used in this study to simulate the viscoelastic effect, which is based on the repulsive force generated by magnetic fields. In the same line of research, the use of this system would strengthen and facilitate the understanding of the role of viscoelasticity in respiratory biomechanics and the analysis of its alterations in healthy and pathological lung conditions, which may enable the use of viscoelasticity as a biomarker.

## Methods

### Magnet characterization

Magnet characterization was performed in a tensile test setup, using an Instron load system 3345 equipped with a 10 N capacity load cell. *Magnet A* was located at the bottom platen of the load system. *Magnet B* was attached to an upper aluminum alloy platen, situated adjacent to the load cell. The arrangement ensured that both magnets confronted the same poles (Fig. 3c). Magnets were located 10 mm away from each other to designate this position as the test baseline. The total vertical displacement achieved by the load cell was 80 mm. Magnets were attached with double-sided tape. Data was acquired at a 50 Hz sampling frequency and was exported and processed in Microsoft Excel.

### Experimental setup for the mechanical ventilation scenario and viscoelastic simulation

A Hamilton-S1 mechanical ventilator was used to establish the mechanical ventilation scenario. This was connected to the alveolar unit of the SAMI-SII respiratory biomechanics simulator, which is the second version of SAMI-S^26^ and was developed in an ongoing doctoral thesis at EIA University, Colombia. The simulator consists of four alveolar units, and each unit is comprised of a pair of bellows, totaling eight bellows overall. For the tests carried out, a single alveolar unit was used and one of its bellows was disabled.

The pneumatic cylinder was placed under the bellow and secured with a series of clamps as Fig. 3b shows, so that both *Magnet A* and *Magnet B* aligned along the same axis. Employing a slide gauge, AB Distance was manually established by vertically adjusting the position of the cylinder. Then, the microcontroller was programmed with the desired PWM value, responsible for governing the velocity of the return stroke. Both the electropneumatic system and the mechanical ventilator were synchronized to operate at identical frequencies. This synchronization enabled the return stroke to occur precisely at the moment of the inspiratory pause during every respiratory cycle. Given that AB Distance was manually set, the development of a control system was not necessary. The cylinder's stroke was 70 mm, constituting the displacement of Magnet A during both extension and return phases. Once the ventilation scenario was started, the electropneumatic system was activated at the moment of an inspiratory pause to synchronize both systems.

The pressure was measured with the Hamilton-S1 mechanical ventilator. During the experiments, data were monitored live and acquired at a 31.25 Hz sampling frequency using a Hamilton Datalogger. After the experiments, data was exported and processed in Microsoft Excel.

### Model parameters calculations and statistical analysis

To find ${E}_{1}$, P_1_-P_2_ pressure drop was calculated for each run within the 2^4^-factorial design as Fig. 6B shows. A D’Agostino-Pearson test was executed to assess the normality of the data distribution. Then, a hypothesis test was performed using a one-sample *t*-test for equal variances with α = 0.05. This facilitated the derivation of the value of ${E}_{1}$ for each distinct AB Distance. To find ${E}_{0}$ and *η*, an exponential regression was done in Microsoft Excel using the paused pressure drops data obtained from the 2^2^-factorial design. The previously found ${E}_{1}$ values were employed as inputs within the regression. Thus, it was possible to find the different values of the parameters of the model for different PWMs and AB Distances (Supplementary Information).

### Supplementary Information

Supplementary Information.

## Data Availability

Data used and/or analyzed during the current study is available from the corresponding author upon reasonable request. Exponential regressions are attached in an Excel file in the supplementary material section.
